# The bacterial potassium transporter gene *MbtrkH* improves K^+^ uptake in yeast and tobacco

**DOI:** 10.1371/journal.pone.0236246

**Published:** 2020-08-17

**Authors:** Baojuan Ding, Xiaoyan Zhang, Yongsheng Xu, Lijia An, Xiangguo Liu, Qiao Su

**Affiliations:** 1 School of Bioengineering, Dalian University of Technology, Dalian, P. R. China; 2 Institute of Agricultural Biotechnology, Jilin Academy of Agricultural Sciences, Changchun, P. R. China; Huazhong University of Science and Technology, CHINA

## Abstract

K^+^ is an essential nutrient for plant growth and is responsible for many important physiological processes. K^+^ deficiency leads to crop yield losses, and overexpression of K^+^ transporter genes has been proven to be an effective way to resolve this problem. However, current research on the overexpression of K^+^ transporter genes is limited to plant sources. TrkH is a bacterial K^+^ transporter whose function generally depends on the regulation of TrkA. To date, whether TrkH can improve K^+^ uptake in eukaryotic organisms is still unknown. In this study, a novel *MbtrkH* gene was cloned from marine microbial metagenomic DNA. Functional complementation and K^+^-depletion analyses revealed that MbTrkH functions in K^+^ uptake in the K^+^-deficient yeast strain CY162. Moreover, K^+^-depletion assays revealed that *MbtrkH* overexpression improves plant K^+^ uptake. K^+^ hydroponic culture experiments showed that, compared with WT tobacco lines, *MbtrkH* transgenic tobacco lines had significantly greater fresh weights, dry weights and K^+^ contents. These results indicate that MbTrkH promotes K^+^ uptake independently of TrkA in eukaryotes and provide a new strategy for improving K^+^-use efficiency in plants.

## Introduction

After nitrogen and phosphorus, K^+^ is one of the main nutrients necessary for all living cells, accounting for 2%-10% of plant dry weight [1]. K^+^ plays a variety of roles in plants, including roles in protein synthesis, osmoregulation, cell expansion, electrical neutralization, photosynthesis, stomatal movement, enzyme activation, salinity stress, starch synthesis and water and nutrient transport through the xylem [1, 2]. K^+^ is abundant in the soil, but the effective K^+^ content available to plants is low [3]. In the absence of sufficient amounts of K^+^, plants produce small seeds and present decreased yields. Therefore, it is important to improve the K^+^ absorption of plants via genetic modification.

The uptake and transport of K^+^ in cells are mediated by several K^+^ transporters, such as transport of K^+^/K^+^ transporter/high-affinity K^+^ transporter (Trk/Ktr/HKT), K^+^ uptake protein/high-affinity K^+^ transporter/K^+^ transporter (KUP/HAK/KT), K^+^ efflux antiporter (KEA), and cation/hydrogen exchanger (CHX) [4–6]. Among these transporters, members of the KUP/HAK/KT family, which is the most widely studied family of K^+^ transporters, are involved in K^+^ transport across membranes in bacteria, fungi, and plants [7]. Currently, KUP/HAK/KT family transporter genes have been cloned in various plant species, including *Arabidopsis thaliana* [8], *Phytolacca acinosa* Roxb [9], *Cryptomeria japonica* [10], *Capsicum annuum* [11], *Alternanthera philoxeroides* [12], *Setaria italica* [13], *Gossypium hirsutum* [14], *Puccinellia tenuiflora* [15], *Manihot esculenta* [16], *Prunus persica* [17], *Lycopersicum esculentum* [18] and mulberry [19]. Several members of the KUP/HAK/KT family have been overexpressed in *Arabidopsis thaliana*, cotton and *Oryza sativa*, and the results indicated that under low-K^+^ conditions, these transporters improved the K^+^ absorption of plants [8, 12, 14]. HKT transporters constitute another important K^+^ transporter family in plants. HKTs can be divided into two different classes on the basis of their Na^+^/K^+^ selectivity [20, 21]. Class 1 HKT transporters, such as AtHKT1;1, OsHKT1;5, and TaHKT1;5, present Na^+^-selective transport activity and have purportedly improve resistance to salinity stress [22–26]. In contrast, class II HKT transporters, such as TaHKT2;1 and OsHKT2;4, function as K^+^-permeable transporters [27, 28]. Compared with KUP/HAK/KT family transporters, HKT transporters provide little case for the improvement of K^+^ absorption efficiency in plants. Although several K^+^-permeable transporters in plants have been functionally characterized, candidate genes for plant K^+^ utilization improvement are still scarce and limited to members of the HAK and HKT gene families.

Most commercially adopted genes have originated from bacteria and provide multiple trait improvement mechanisms from bacteria into crop plant species [29]. Many genes involved in insect resistance (cry1A, cry2A, cry1F, cry3A, cry3B, cry34A, cry35A, and vip3A and their variants) have been identified from *Bacillus thuringiensis* (Bt) and have been introduced into commercial transgenic crops to provide resistance to lepidopteran and coleopteran pests [30]. The genes that encode the phosphinothricin acetyltransferase (PAT) and 5-enolpyruvylshikimate-3-phosphate synthase (EPSPS) enzyme variants that confer glufosinate and glyphosate tolerance to plants, respectively, were also cloned from bacteria [30]. The first commercial drought tolerance maize included the MON 87460 event, in which cold-shock protein B (CSPB) cloned from *Bacillus subtilis* was expressed [31, 32]. Exploring new K^+^ transporter genes from bacteria is expected to be a new way to improve plant K^+^ use.

In *Escherichia coli*, Trk K^+^ is the major constitutive K^+^ transporter [33, 34]. The Trk K^+^ transporter system in bacteria comprises two parts: the transmembrane protein TrkH and the regulatory protein TrkA. In general, in bacteria, TrkH depends on the regulation of TrkA. Interestingly, a recent search revealed that TrkA is not necessary in some bacteria when the expression of TrkH is high, and a similar phenomenon was also observed in fungi [4]. It was speculated that TrkH can function as a K^+^ transporter in eukaryotic organisms independently of TrkA, and there may be other mechanisms that provide energy or regulate the activation in complex with the eukaryotic system [33,35]. However, to date, fundamental questions regarding whether TrkH improves K^+^ transport as an ion channel in the absence of TrkA expression in plants remain unresolved.

In the present study, a novel *trkH* gene, named *MbtrkH*, was cloned from marine microbial metagenomic DNA via degenerate PCR and anchored PCR, and then the function of *MbtrkH* in mediating K^+^ uptake was confirmed by the use of yeast functional complementation experiments. *MbtrkH* was subsequently introduced into tobacco via *Agrobacterium*-mediated leaf disc transformation, and the K^+^ transport function of MbTrkH was verified by K^+^-depletion and K^+^-supplementation hydroponic culture experiments. Our results reveal that MbTrkH can function as a K^+^ transporter in eukaryotic organisms independently of TrkA and that overexpression of *MbtrkH* is an efficient way to enhance K^+^-use efficiency in plants.

## Materials and methods

### Marine microorganisms, strains, media and growth conditions

Seawater samples were collected from the vicinity of the Black Rock reef, Dalian, Liaoning Province. The seawater samples were filtered, and microorganisms were retained on the filter membrane. Clean blades were then used to scrape the membrane gently, after which the mixed samples of marine microorganisms were collected, washed with seawater and then centrifuged at 8000 rpm for 2 min. The samples were stored and used in subsequent DNA extraction experiments. Yeast (*Saccharomyces cerevisiae*) strain CY162 [*MATa*, *△trk1*, *trk2*::*pCK64*, *his3*, *leu2*, *ura3*, *trp1*, *ade2*] [36] is a K^+^-uptake-defective mutant and was incubated in yeast peptone dextrose (YPD) media supplemented with 50 mM K^+^ (pH 7.5) at 28°C. Synthetic complete (SC)-uracil (Ura) media containing 20 g·L^-1^ galactose (gal) and 50 mM K^+^ were used for yeast functional complementation experiments. *Escherichia coli* strain 10407 was grown in Luria-Bertani (LB) media (pH 7.5) at 37°C.

### Plant material and stress treatments

Seeds of tobacco were surface sterilized and sown on 1/2-strength Murashige-Skoog (MS) [37] solid media. After one month, uniform seedlings were transferred to a 1/2-strength Hoagland nutrient solution [38] and then allowed to grow for another 2–4 weeks before stress treatments. Modest aeration was provided, and the pH was adjusted to 6.0; the solution was replaced every 3 d. The greenhouse was maintained under a 16-h light (28°C)/8-h dark (22°C) photoperiod, and the relative humidity was maintained at 60%-70%.

With respect to K^+^-depletion experiments, tobacco seedlings were pre-treated in a K^+^-free nutrient solution in which KNO_3_ and KH_2_PO_4_ were replaced with Ca(NO_3_)_2_ and NaH_2_PO_4_, respectively, at 22°C for 2 d and then transferred to depletion solution (1 mM KCl and 0.2 mM CaSO_4_). Samples of the solution were collected every 12 h, and the depletion period lasted 72 h. The K^+^ concentrations in the samples were subsequently measured by an atomic absorption spectrophotometer (Thermo Scientific, Shanghai, China).

With respect to low-K^+^ hydroponic culture assays, tobacco seedlings of similar size were transferred to K^+^-deficient (LK, 1 mM) solution and 1/2-strength Hoagland nutrient (normal, 3 mM) solution. KCl was added to a K^+^-free nutrient solution, which was adjusted to 1 mM for the K^+^ deficiency experiments. All experiments included three independent biological replicates.

### Marine microbial metagenomic DNA isolation and gene cloning

Routine DNA isolation was carried out according to standard procedures [39]. The quality of DNA was determined via gel electrophoresis in conjunction with a Nanodrop 2000c spectrophotometer. A search of cloned *trkH* homologues was performed with the BLAST program. According to the conserved sequences of these TrkHs, two degenerate primers were designed to amplify the conserved sequences of *trkH*, and the resulting PCR products were purified and sequenced. Both the 5’ and the 3’ ends of the *trkH* gene from marine microbial metagenomic DNA were cloned via anchored PCR. Primers were designed according to the sequence obtained previously. Fragments approximately 650 bp (for the 5’ end) and 200 bp (for the 3’ end) in length were purified and sequenced. A putative open reading frame (ORF) was identified by the ORF finder program of the NCBI, and full-length DNA of *MbtrkH* was obtained by the use of ORF amplification primers. The relevant primers used are listed in S1 Table. Transmembrane regions and conserved domains were predicted by TMpred and the Conserved Domain Search Service, respectively. A homologue search was performed using the BLAST program, and a phylogenetic tree of TrkH was subsequently constructed via ClustalX, BioEdit and MEGA 4.0. The ORF of *EctrkH* was cloned by the use of *Escherichia coli* DNA as a template together with primers designed according to gene sequence information within the NCBI database.

### Construction of vectors

The ORF of *MbtrkH* was cloned into the *EcoRI* and *SphI* sites of a pYES2.0 vector for expression in yeast strain CY162 and into the *SmaI* and *SpeI* sites of a pTF101 vector for expression in tobacco. *MbtrkH* was further subcloned into the *SacI* and *XbaI s*ites of a pCAMBIA2300-GFP vector for subcellular localization experiments. DNA sequencing indicated successful ligation into the expression vector.

### Functional complementation analysis and K^+^-depletion assays in yeast

To test the role of the protein encoded by *MbtrkH* in K^+^ uptake, the yeast strain CY162, which lacks K^+^ uptake system activity, was used for growth tests and cation uptake experiments. Chemically competent CY162 cells were prepared and transformed with a pYES2.0 empty vector, pYES2.0-*EctrKH* or pYES2.0-*MbtrkH* as described above. The transformed cells were cultured in SC-Ura media at 28°C for 3 d. Individual colonies were subsequently selected and cultured in YPD liquid media consisting of 50 mM K^+^. One millilitre of the overnight culture was inoculated into 100 mL of fresh SC-Ura containing gal and consisting of 50 mM K^+^ and subsequently cultured for 48 h. The culture was then washed and re-suspended in SC-Ura containing gal and consisting of 3 mM K^+^, and the OD_600_ was adjusted to 1. The cultures were serially diluted and then spotted on SC-Ura solid media containing gal and consisting of 3 or 50 mM K^+^ and subsequently incubated at 28°C for 4 d.

With respect to K^+^-depletion assays, yeast strains were cultured in K^+^-free SC-Ura media for 5 h, after which K^+^-starved yeast strains were suspended in SC-Ura containing gal and consisting of 3 mM K^+^; the concentration of yeast cells was subsequently adjusted to an OD_600_ of 0.4. Samples were taken at intervals of 1 h, and the K^+^ concentration in the depletion solution was determined by atomic absorption spectrophotometry.

To determine the growth of yeast at different K^+^/Na^+^ concentrations, yeast strains cultured in K^+^-free SC-Ura media were streaked on solid media supplemented with K^+^ to final concentrations of 0.1, 0.5, 1, 3, or 50 mM and then incubated at 28°C for 4 d. Correspondingly, yeast cells were streaked on SC-Ura media consisting of different concentrations of NaCl (0, 50, 100, 200, or 500 mM) and 3 or 50 mM K^+^ separately to test the influence of Na^+^ on the growth of transformed yeasts.

### Genetic transformation and molecular detection of tobacco plants

The vector construct was transferred into *Agrobacterium tumefaciens* strain EHA101. Tobacco (*Nicotiana tabacum*) plants were subsequently transformed via the *Agrobacterium-*mediated leaf disc transformation method [40]. Independent T0 generation transgenic lines were screened on 1/2-strength MS media consisting of 1.5 mg·L^-1^ bialaphos and verified by PCR analysis.

Tobacco genomic DNA was extracted from young leaves of wild-type (WT) and transgenic plants via a DNA Quick Plant System (Tiangen, Beijing, China). Total RNA was isolated via TRIzol reagent (Invitrogen) according to the manufacturer’s instructions. First-strand complementary DNA (cDNA) was synthesized via a PrimeScript^TM^ RT Reagent Kit (TaKaRa, Beijing, China) according to the manufacturer’s instructions. The cDNA was used as a template for PCR, and the PCR amplification conditions were as follows: 94°C for 5 min; 27 cycles of 94°C for 30 s, 58°C for 30 s, and 72°C for 1 min; and a final extension at 72°C for 10 min. As a control for equal cDNA amounts in each reaction, PCR was performed with the *Actin* gene. The relevant primers used are listed in S1 Table.

### Phenotype, fresh weight, dry weight and K^+^ content of seedlings

After 21 d of stress treatment, tobacco seedlings were harvested, and the plant phenotypes were observed. The seedlings were rinsed in distilled water, soaked, and then weighed to obtain their fresh weight. The harvested seedlings were separated into shoots and roots, dried in an oven at 105°C for 30 min, and then dried at 80°C to a constant weight to determine their dry weight.

To determine the K^+^ concentration, the dried roots and shoots were ground into a fine powder, weighed, and then fully digested with HNO_3_:HClO_4_ (4:1) for elemental extraction. The concentrations of K^+^ in appropriately diluted samples were determined via an atomic absorption spectrophotometer (Thermo Scientific, Shanghai, China).

### Subcellular location of MbTrkH in tobacco

Co-infiltration of tobacco leaves with pCAMBIA2300*-MbtrkH-eGFP* and a plasma membrane marker vector (pm-rk CD-1007 fused to mCherry) [41] was performed as described previously, with minor modifications [42]. GFP and mCherry signals were observed by the use of a Leica SP8 confocal microscope, and the images were processed with Adobe Photoshop 7.0.

### Statistical analysis

The data of the WT and transgenic lines were compared, the details of which are shown in the figure legends. Graphs were produced via Origin 9.0 software. P values were generated using Student’s t test to indicate significant differences between the WT and transgenic lines.

## Results

### Cloning the *MbtrkH* genes

We cloned a novel *trkH* gene from marine microbial metagenomic DNA, named *MbtrkH*. The protein encoded by *MbtrkH* was named MbTrkH. The ORF of the *MbtrkH* gene was 1392 bp long and encoded 463 amino acids (accession number: MK863632). MbTrkH was predicted to contain 10 transmembrane domains and belonged to the TrkH superfamily. The amino acid sequence of MbTrkH was more than 59% homologous to that of *Exiguobacterium*. A phylogenetic tree indicated that MbTrkH was distantly related to and members of the currently verified TrkH K^+^ transporter family. Moreover, we cloned *EctrkH* from *Escherichia coli* as a reference (accession number: NC000913).

### MbTrkH mediates K^+^ uptake in yeast cells

**Fig 1:**
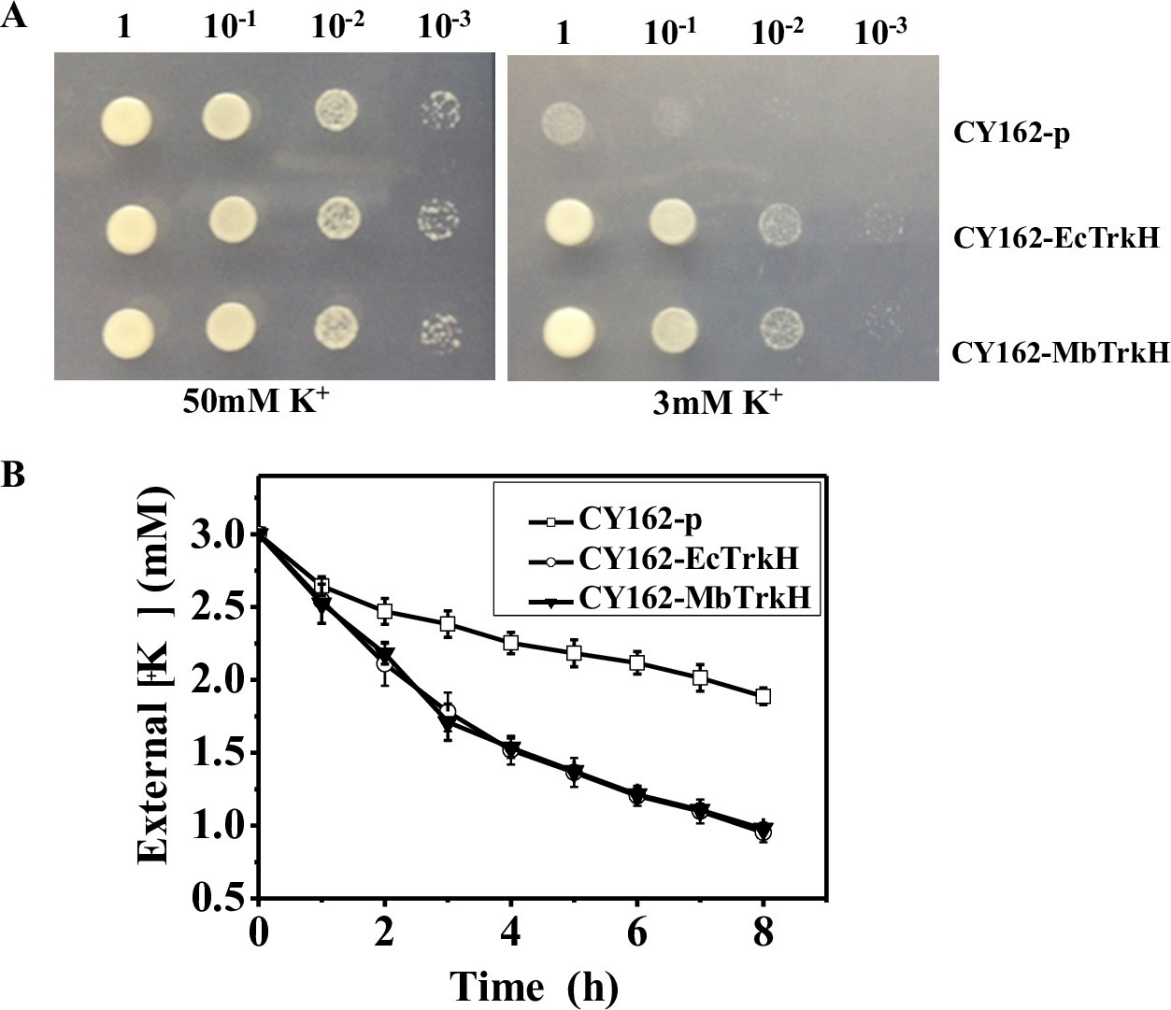
Complementation and depletion analyses of the K^+^-uptake-defective *Saccharomyces cerevisiae* strain CY162 harbouring MbTrkH (CY162-MbTrkH), EcTrkH (CY162-EcTrkH), or an empty vector (CY162-p). (A) Yeast complementation assay. All transformed cells were grown on SC-Ura media that lacked Ura. The yeast was serially diluted 10-fold and spotted on media supplemented with 50 mM K^+^ or 3 mM K^+^ and cultured for 4 d. The empty vector was used as a negative control. (B) K^+^-depletion assays of yeast under 3 mM K^+^. K^+^-starved cells were suspended in depletion solution. Samples were taken every 1 h, and then the same volume of KCl (3 mM) was added to maintain the total volume of the depletion solution. The K^+^ concentration in the depletion solution was measured via atomic absorption spectrophotometry. The following are shown: the empty vector (open squares), EcTrkH (open circles), and MbTrkH (open triangles).

The pYES2.0 plasmid (empty vector) and the pYES2.0 plasmids containing *EctrkH* and *MbtrkH* were transformed into yeast strain CY162, which is an endogenous K^+^-uptake-defective yeast strain; the three transformed yeast strains were defined as CY162-p, CY162-EcTrkH and CY162-MbTrkH, respectively. The results of complementation experiments showed that all transformed yeast strains grew well in the presence of 50 mM K^+^ (Fig 1A) and that the CY162 strains containing *MbtrkH* or *EctrkH* grew normally, while the strain containing the empty vector was not able to grow when subjected to 3 mM K^+^ (Fig 1A). Yeast K^+^-depletion experiments showed that the CY162 yeast harbouring MbTrkH and EcTrkH were able to deplete external K^+^ concentrations to values less than 1 mM, while the yeast harbouring the empty vector depleted external K^+^ concentrations to values greater than 2 mM after 8 h of depletion (Fig 1B).

### Functional analysis of yeast under different K^+^ and Na^+^ concentrations

**Fig 2:**
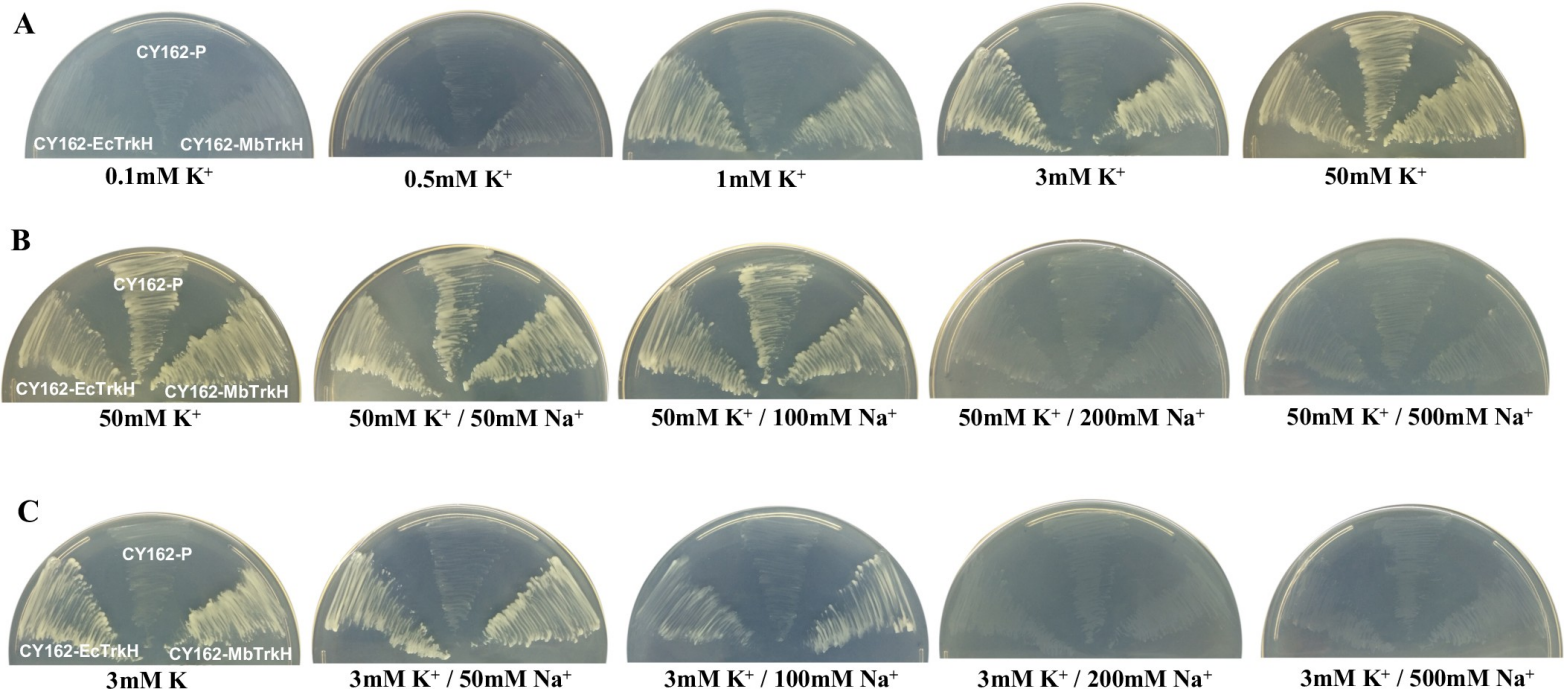
Growth experiments of cells of K^+^-uptake-defective *Saccharomyces cerevisiae* CY162 strain harbouring MbTrkH (CY162-MbTrkH), EcTrkH (CY162-EcTrkH), or an empty vector (CY162-p) under different K^+^ and Na^+^ concentrations. (A) Growth status of yeast under different K^+^ concentrations. The yeasts were inoculated in SC-Ura media consisting of 0.1, 0.5, 1, 3 or 50 mM K^+^ and cultured for 4 d. (B) Growth status of yeast under 50 mM K^+^ at different Na^+^ concentrations. (C) Growth status of yeast under 3 mM K^+^ at different Na^+^ concentrations. The yeast cells were incubated in SC-Ura media supplemented with 0, 50, 100, 200 or 500 mM Na^+^ and cultured for 4 d for B and C.

To analyse K^+^ transport mediated by MbTrkH and EcTrkH further, K^+^-uptake experiments were performed with different K^+^ concentrations. When the K^+^ concentration was 0.1 mM, the three transformed CY162 strains did not grow (Fig 2A). However, yeasts expressing MbTrkH or EcTrkH grew better than the yeast harbouring the empty vector under K^+^-limited conditions, whose concentrations were 0.5, 1 and 3 mM (Fig 2A). The growth of CY162 cells harbouring MbTrkH or EcTrkH was similar to that of the cells harbouring the empty vector in the presence of 50 mM K^+^ (Fig 2A). To study the effects of NaCl on yeast growth, the three transformed CY162 strains were subjected to different NaCl treatments: 50 mM K^+^ or 3 mM K^+^. The growth of the three yeast strains in the media consisting of 0, 50 and 100 mM Na^+^ was essentially the same (Fig 2B), while the growth was significantly inhibited under 200 and 500 mM Na^+^ (Fig 2B). Under 3 mM K^+^ conditions, the three transformed yeast strains grew differently. When the media consisted of 0, 50 or 100 mM Na^+^, the growth of the yeast strains harbouring MbTrkH or EcTrkH was similar and better than that of the yeast strain harbouring the empty vector. Moreover, the yeast harbouring MbTrkH grew slightly better than the yeast harbouring EcTrkH when the media consisted of 100 mM Na^+^ (Fig 2C). When the media consisted of 200 or 500 mM Na^+^, the growth of the three yeast strains was significantly inhibited (Fig 2C).

### MbTrkH improved K^+^ absorption in transgenic tobacco lines

**Fig 3:**
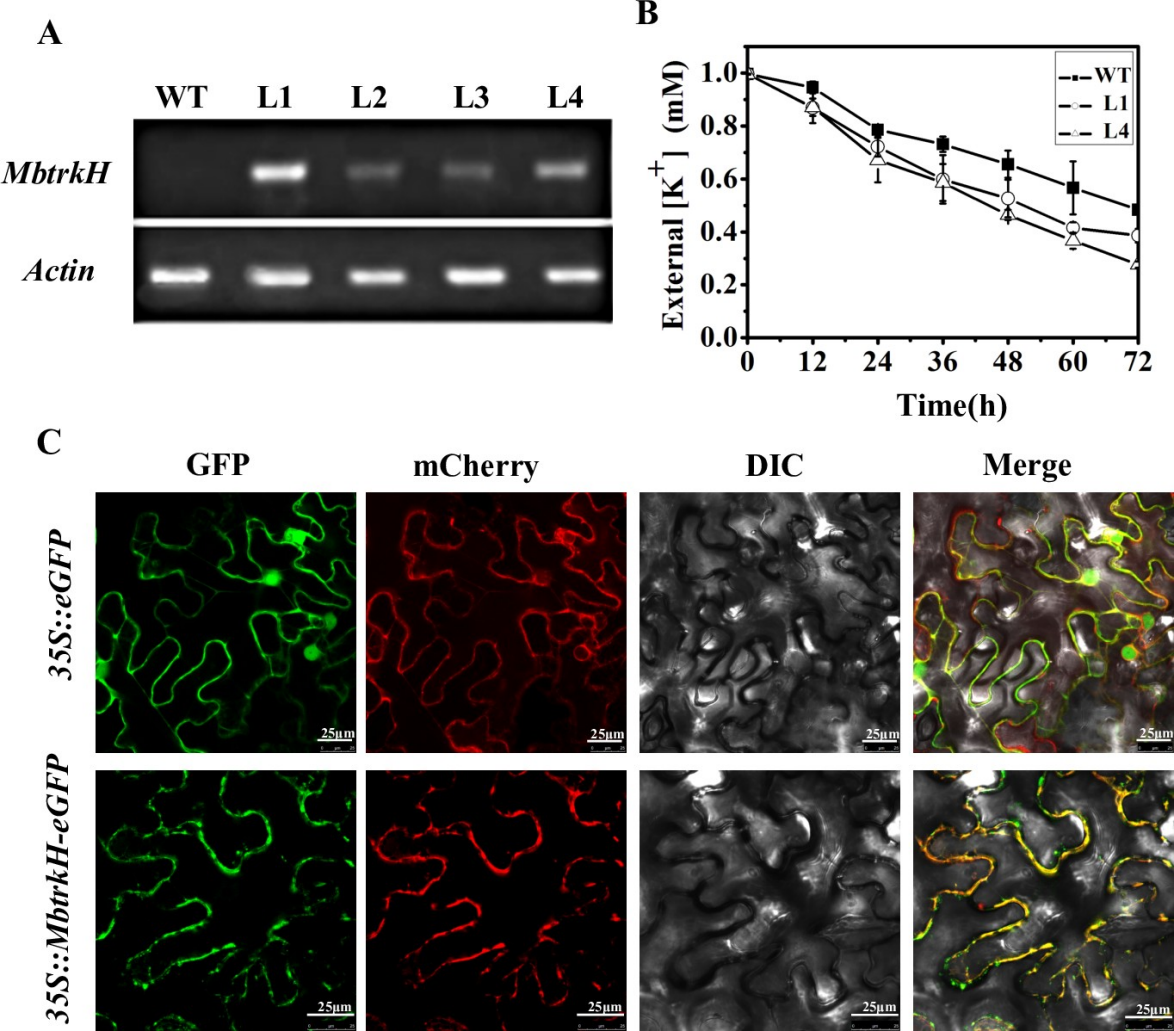
Functional verification of *MbtrkH* by transformation analysis. (A) Expression of *MbtrkH* in transgenic plants (lines 1–4) and controls (CK) as revealed by RT-PCR. (B) Tobacco K^+^-depletion assay under 1 mM K^+^. WT is the wild-type line; L1 and L4 are two independent *MbtrkH* transgenic lines. Samples were taken every 12 h for 72 h. The oxygen supply was maintained for 24 h during the treatment period. The K^+^ concentration was measured via atomic absorption spectrophotometry. (C) A pCAMBIA2300*-35S*::*MbtrkH-eGFP* construct was used to assess protein localization. A pCAMBIA2300-35S::*eGFP* construct was used as a control, and a pCAMBIA2300-35S::*pm-rk-mCherry* construct was used as a plasma membrane-positive marker. Positive markers were co-transformed together with pCAMBIA2300-35S::*MbtrkH-eGFP* and pCAMBIA2300-35S::*eGFP* in each independent transformation. MbTrkH-GFP was detected in the plasma membrane of tobacco leaf epidermal cells. GFP images are shown under dark field to indicate GFP fluorescence, and mCherry images are shown under dark field to indicate mCherry fluorescence. Differential interference contrast (DIC) images were taken under bright light to visualize cell morphology. The merge images were formed by merging the GFP, mCherry and DIC images. Bar = 25 μm.

To assess the function of MbTrkH in plants further, we generated transgenic *MbtrkH*-overexpressing tobacco plants. A pTF101*-MbtrkH* vector containing the CaMV35S promoter driving the transcription of *MbtrkH* was constructed and subsequently transformed into tobacco. The transgenic plants were then tested via PCR and semi-quantitative RT-PCR with gene-specific primers, and four independent transgenic lines were obtained. The L1 and L4 tobacco lines with relatively high transcript levels of *MbtrkH* were chosen for subsequent physiological experiments (Fig 3A). To evaluate whether MbTrkH improved the K+ absorption capability of transgenic tobacco lines under low-K^+^ conditions, we performed K^+^-depletion tests under 1 mM K^+^. The L1 and L4 transgenic lines displayed greater K^+^ uptake capabilities than did the WT plants after 72 h of depletion (Fig 3B). These results show that overexpression *of MbtrkH* can improve K^*+*^ absorption in transgenic tobacco.

For subcellular localization studies, a pCAMBIA2300*-MbtrkH-eGFP* vector containing the CaMV35S promoter driving transcription of the MbTrkH-eGFP fusion protein was constructed and used for subsequent transient expression in tobacco. We co-transformed the pCAMBIA2300*-MbtrkH-eGFP* vector and a plasma membrane localization marker (pm-rk CD-1007 fused to mCherry) into tobacco leaf epidermal cells [41]. Confocal microscopy analysis revealed that the MbTrkH-eGFP signal and the positive marker co-localized well, with both detected in the plasma membrane of leaf epidermal cells (Fig 3C). These results indicate that MbTrkH is targeted mainly to the plasma membrane, which is consistent with previous findings that K^+^ transporters are localized in the cell membrane [25–28,43].

### The plant phenotype, fresh weight, dry weight and K^+^ content improved in *MbtrkH* transgenic tobacco lines

**Fig 4:**
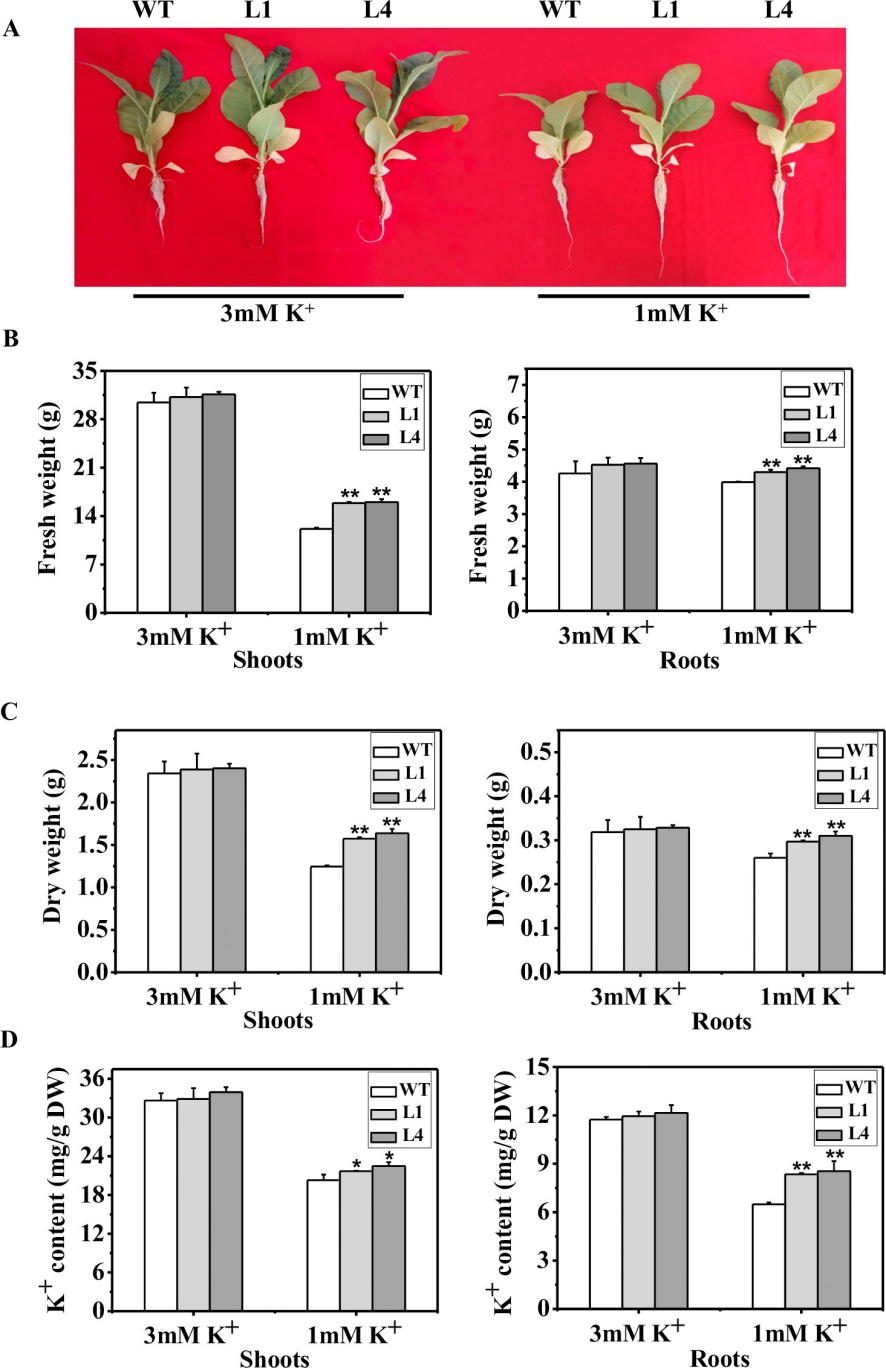
Analysis of the phenotypes, fresh weight, dry weight and K^+^ content of *MbtrkH* transgenic plants. (A) Phenotypes of transgenic lines and WT plants under 3 mM K^+^ or 1 mM K^+^ after 21 d. (B) Plant fresh weight. (C) Plant dry weight. (D) Plant K^+^ content. L1 and L4 are two independent *MbtrkH* transgenic lines; WT is the wild-type line. The error bars represent the standard errors (n = 6). Differences between two groups of data for comparisons in all of the experiments in this study were considered statistically significant (*,*p*< 0.05) or extremely significant (**, *p* < 0.01).

To assess whether the overexpression of *MbtrkH* influenced plant growth and development further, the seedlings were cultured in nutrient solution supplemented with 1 or 3 mM K^+^ for 21 d. In the presence of 3 mM K^+^, there was no difference in phenotype, fresh weight, dry weight or K^+^ content between the transgenic lines and WT plants (Fig 4A–4D). The growth of both the transgenic lines and WT plants was inhibited when the plants were cultured in the presence of 1 mM K^+^, but compared with the WT plants, the transgenic lines were taller and displayed greater root lengths, fresh weights, dry weights and K^+^ contents (Fig 4A–4D). Our results support that the overexpression of *MbtrkH* improves plant growth and development by enhancing K^+^-use efficiency.

## Discussion

K^+^ is the most abundant cation in plant cells and plays a vital role in many fundamental processes, including plant growth and development [44, 45]. K^+^ deficiency affects crop yield and quality [46], and overexpression of K^+^ transporter genes is an effective way to resolve these issues [47]. However, K^+^ transporter genes are mainly restricted to plant sources [13, 14]. TrkH is a bacterial K^+^ transporter whose function generally depends on the regulation of TrkA [4]. Although the structure and function of TrkH have been thoroughly studied in bacteria, the function TrkH in eukaryotic organisms is still unknown [4, 35].

In this study, the *MbtrkH* gene was cloned from marine microbial metagenomic DNA and introduced into the K^+^-deficient yeast strain CY162. Functional complementation and K^+^-depletion experiments revealed that the *MbtrkH* gene function in K^+^ absorption in yeast (Fig 1A and 1B). Yeasts harbouring MbTrkH or EcTrkH grew better than those harbouring the empty vector when the K^+^ concentration was greater than 0.5 mM, indicating that MbTrkH is a low-affinity K^+^ transporter (Fig 2A), which is consistent with its function in many bacteria [48–50]. Under 3 mM K^+^ and 100 mM Na^+^ conditions, yeast harbouring *MbtrkH* grew slightly better than yeast harbouring *EctrkH* (Fig 2C), the results of which are similar to those of *AatrkH*. *AatrkH* was cloned from bacterial samples from a soda lake and introduced into K^+^-uptake-defective *Escherichia coli* LB2003; functional complementation assays revealed that *Escherichia coli* harbouring *AatrkH* grew slightly better than did *Escherichia coli* harbouring *EctrkH* under 3 mM K^+^ and 115 mM Na^+^ conditions [51].

To test whether the *MbtrkH* gene functions in K^+^ uptake in plants further, the *MbtrkH* gene was transferred into tobacco plants. K^+^-depletion experiments subsequently indicated that the *MbtrkH* gene improved the K^+^ uptake capability of the transgenic plants (Fig 3). In addition, K^+^ hydroponic culture experiments showed that overexpression of *MbtrkH* significantly increased the K^+^ content in plant tissues and thus increased the height, root length and biomass of the transgenic plants (Fig 4), which was consistent with the results for *GhKT2*. Overexpression of *GhKT2* increased plant K^+^ uptake, dry weight and the K^+^ content in *Arabidopsis* [14].

In summary, the results of yeast complementation and K^+^-depletion experiments indicated that the bacterial *MbtrkH* gene functions in K^+^ absorption independently of TrkA in yeast. Afterward, the *MbtrkH* gene was transferred into tobacco plants via the *Agrobacterium*-mediated leaf disc transformation method. The results of K^+^-depletion and hydroponic culture experiments showed that *MbtrkH* functions in K^+^ uptake in plants. Several K^+^ transporter genes have been overexpressed and have been shown to improve the K^+^ absorption capability of plants, but these genes are limited to plant sources. The results presented here indicate that bacterial *MbtrkH* might be a potential candidate gene for the genetic modification of crops to improve K^+^ nutrition.

## Supporting information

S1 Table Primers used in this work. (PDF)
