# Supplementary material for: The bacterial potassium transporter gene MbtrkH improves K+ uptake in yeast and tobacco
Source: PLoS One. 2020 Aug 17;15(8):e0236246. doi: 10.1371/journal.pone.0236246 (PMC7430745; doi:10.1371/journal.pone.0236246)
Supplement: S1 Table — (PDF) [file pone.0236246.s001.pdf]

Supplementary Material

Table S1. Primers used in this work

Purpose	Primer (5’-primer end to 3’-primer end)
Degenerated primer pairs, partial DNA cloning	F: AAYAATGCKGGYTTYGCMYTATG
	R: ACRCGTCCGAGTCATRAACATGAT
3’- end nested PCR amplification	out: GGCAGGGTTCCAGACGATTGATTTG
	in: AGTCACGACAGCAGCCGTCATCTTA
5’- end nested PCR amplification	out: GAAAGTGGCACCATCGTCGTCAAAT
	in: ATTTTGAAGGGAACCGAGATGTGAG
Anchored primer	Oligo dC: CCCCCCCCCCCCCCCC
MbtTkH ORF amplification	F: ATGTTTTCGCATAAAAGACGTAC
	R: TTACCCGATCAGCATATTTTCTTTC
EcTrkH ORF amplification	F: CGGAATTCATGCATTTTCGCGCCATTACC
	R: GGGCTGAAAGACCGCACTTACTAGATCTCG
PCR detection of *MbtrKH* gene	F: ATGTTTTCGCATAAAAGACGTAC
	R: TTACCCGATCAGCATATTTTCTTTC
PCR detection of *Actin* gene	F: CTATTCTCCGCTTTGGACTTGGCA
	R: AGGACCTCAGGACAACGGAAACG

For degenerate primers in this study, Y = C or T, K = G or T, and R = A or G.
